# Medication Error Rate in Transition of Care: General Practitioner (GP) Referrals to a Regional Emergency Department

**DOI:** 10.3390/healthcare7040152

**Published:** 2019-11-28

**Authors:** Sarah J. Prior, Colleen Cheek, Dong Cheah, Christopher Etherington, Abigail Williams, Nicole S. Reeves

**Affiliations:** 1 School of Medicine, College of Health and Medicine, University of Tasmania, Burnie 7320, Tasmania, Australia; nicole.reeves@utas.edu.au; 2 Tasmanian Health Service, North West Regional Hospital, Burnie 7320, Tasmania, Australia; colleen.cheek@utas.edu.au (C.C.); abigail.williams@ths.tas.gov.au (A.W.); 3 Rural Clinical School, College of Health and Medicine, University of Tasmania, Burnie 7320, Tasmania, Australia; cheahdonglum@gmail.com (D.C.); christopher.etherington95@gmail.com (C.E.)

**Keywords:** medication, general practice, discrepancy, pharmacist, risk, referral, best practice medication history

## Abstract

Medication errors have a significant impact on patient outcomes, increase healthcare costs, and are a common cause of preventable morbidity. This single-site, observational, diagnostic accuracy study aimed to quantify medication discrepancies in transition of care from primary care to the emergency department (ED) over a 12-month period. Medication lists in General Practitioner (GP) referrals to a regional ED were examined against a Best Possible Medication History (BPMH) performed by a hospital pharmacist. One hundred and forty-three patients (25%) with computer-generated GP referrals to ED who were subsequently admitted to hospital had a BPMH taken; 135 (94%) of these had at least one medication discrepancy identified with a discrepancy rate of 67.18 discrepancies per 100 medications. Improving medication reconciliation in the community may reduce the burden associated with preventable medication errors. Whether this is achieved by more frequent GP-led medication review or community-based pharmacist medication review may depend on the community and available resources.

## 1. Introduction

Medication errors have health and economic consequences for patients and health services. It is estimated that 2%–3% of all hospital Australian admissions are medication-related [1], with 66%–75% of patients having a medication error at time of admission [2,3] to an Australian hospital, 30% of which have potential to cause harm [4,5]. A recent review conducted by the United Kingdom (UK) National Health Service (NHS) found errors occurred at all stages of medication use, from prescribing and dispensing, through administration and monitoring, and they occurred in primary care (38.3%), care homes (41.7%), and secondary care (20%) [6]. Errors in primary care may contribute greatly to the burden on health systems due to the size of the sector, with the impact estimated to cost the NHS £98.5 million per year, consuming 181,626 bed-days, causing 712 deaths, and contributing to 1708 deaths during hospitalisation [6]. The authors suggest UK rates are similar to those in comparable settings. There were, however, few quality studies of error rates during transition of care and most studies pertained to medication errors for patients discharged from hospital care.

Best practice medication management includes reconciling medicines on admission to a health service. A Best Possible Medication History (BPMH) involves a structured patient interview and confirmation with at least one other source and is often undertaken by the hospital pharmacist for admitted patients. High-risk medications include the anti-infectives, potassium and other electrolytes, insulin, narcotics and other sedatives, chemotherapeutic agents, and heparin and other anticoagulant (APINCH) medicines ([7]. Using the BPMH to compare with the medications listed in General Practitioner (GP) referrals, a multisite study conducted in Australian hospitals in 2008–2009 found 87% of GP referrals had one or more discrepancies in the patients’ regular medications and 62% had one or more regular medication discrepancies of moderate to high significance [8].

Computerised medication systems or some computerised functionality is regarded as a key component of safer medication management. The purported benefits include auto population of medications into transfer of care documents such as referrals, minimising errors associated with ‘cutting and pasting’, up-to-date and accurate lists, inclusion of new, suspended or changed medications supporting clinical handover communication, support of workflow that prompts medication review when patients are identified as a falls risk, and alerts for prescribers when prescribing look-alike, sound-alike and high-risk medications [7]. An accurate and complete current medication list is a key component of transfer of care documents such as referrals, discharge summaries, and the shared health summary (SHS) that is a crucial part of the Australian electronic MyHealth record system. A SHS is particularly recommended for patients with chronic medical conditions or following a 75+ health assessment and may be created and uploaded by a medical practitioner, usually the patients’ GP, a registered nurse, or an Aboriginal and Torres Strait Islander Health Practitioner [9]. With increased focus over the last decade on computerised systems including the MyHealth record to provide the right information, for the right patient, at the right time, we sought to quantify the medication discrepancies for patients referred to an emergency department (ED) who were subsequently admitted to hospital and had a Best Possible Medication History taken by a pharmacist. The overarching aim of this study was to identify where strategies to reduce persisting medication error might be best targeted.

## 2. Materials and Methods

This was a single-site, observational, diagnostic accuracy study undertaken for patients presenting to the ED of an Australian regional hospital between 1 June 2015 and 30 May 2016. Ethics approval was obtained from the Tasmanian Health and Medical Research Ethics Committee (Ref: H0015862).

Patient records were identified retrospectively from the ED medical information system as being referred to the ED by a GP and subsequently admitted as an inpatient. For each patient record, a medication list provided by the GP was compared to the BPMH taken by the hospital pharmacist. The BPMH did not include any medications commenced in the ED as a discrepancy. Medication discrepancies were recorded as omissions, false inclusions, dose and frequency errors, route of administration error, class discrepancies and dosage or frequency omissions.

**Figure 1:**
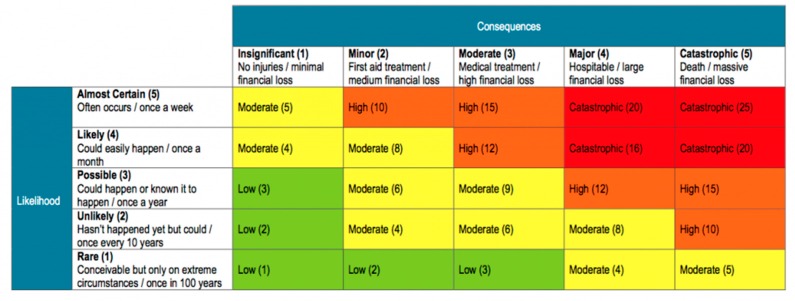
Risk assessment matrix.

Each discrepancy was given a risk rating by calculating the consequence and likelihood of occurrence using a Risk Assessment Matrix (Figure 1). The severity of the consequences was assessed by an emergency medicine clinician and rated as insignificant, minor, moderate or major. The likelihood of occurrence was assessed by an emergency medicine clinician and was rated as rare, unlikely, possible, likely, or almost certain.

## 3. Results

A total of 563 patient records were scrutinised with 143 (25%) of these having both a GP referral and a best possible medication history. Of the 143 patient records, 74 were males and 69 females. A total of 135 (94%) of these histories contained at least one medication discrepancy, all of whom attended ED with computer-generated GP referrals. Inclusions were the most common discrepancy (40%) with omissions being the next most frequent (24.7%) (Table 1). The rate of medication error (number of medication discrepancies per 100 medications) was 67.18.

### 3.1. Consequences

Following clinical evaluation of the medication discrepancies, it was found that 94 (15.3%) of discrepancies were of moderate to major consequence (Table 2). The eight discrepancies of major consequence were seen in three patients. One patient had 13 discrepancies, of which three were of major consequence. One patient had five medications listed on their referral, none of which the patient was taking; the four medications not listed that the patient was found to be taking in the BPMH included an novel oral anticoagulant (NOAC) and a steroid. One patient had one medication listed in the GP referral the patient was not taking, whereas the BPMH found the patient was taking six unlisted medications, including Gliclazide and Metformin. The most common drugs associated with discrepancies of major consequence were insulin and another diabetic medication.

### 3.2. Likelihood

The likelihood of consequences occurring was almost certain or likely in 28 (4.6%) instances (Table 3).

### 3.3. Risk

The consequence and likelihood data were utilised to determine a risk rating for the medication discrepancies. The majority (72.9%) of discrepancies were low risk (Table 4). However, the risk was high or extreme in 96 (15.6%) instances.

The 13 extreme risk ratings were from discrepancies of major consequence which were likely (*n* = 7) or almost certain (*n* = 1) and applied to five patients. One patient presented with hypercalcaemia and the GP referral listed five diabetic medications the patient was not taking, and was missing the apixaban, cholecalciferol, magnesium and prednisone the patient was taking; one patient presented with abdominal pain, tachycardia, and atrial fibrillation and the referral contained one medication the patient was not taking, and was missing six medications the patient was taking; one patient presented with bilateral pulmonary infiltrate for whom the supplements fish oil, glucosamine and magnesium were missing from the GP referral; one patient presented with abdominal pain whose blood pressure medication was not recorded on the GP referral list nor were two medications for depression. In only one of these instances was the referring GP not the patient’s usual GP.

The 78 high-risk ratings related to 35 patients, two of whom also had extreme ratings for some discrepancies. Of the remaining patients, one patient presenting with a diabetic foot ulcer had eight discrepancies rated as high risk, none of the 11 patient’s medications were contained in the referral; one patient presenting with shortness of breath and chest pain had three medications listed, only one of which the patient was taking, together with nine others missing in the referral, including a nitrate, nitrate spray and a vasodilator. In both instances, the referring GP was the patient’s usual GP.

In 48.8% of the high-risk discrepancies the GP authoring the referral was listed as the patients usual GP, and no one GP was represented more than another.

## 4. Discussion

Medication errors have the potential for significant patient harm and cost to the health system. Computerised medication management is promoted to reduce the likelihood of error. In this study, the rate of medication discrepancies observed in General Practitioner (GP) patient referrals where at least one medication discrepancy occurred was 67% despite the use of computerised systems for medication management. One-quarter of these discrepancies were of a moderate to extreme risk.

The premise of having a nominated provider for the electronic health information summary (Australia), MyHealth record, is that it is critical to have shared health summaries (SHSs) that are clinically useful and effective for a range of different types of healthcare providers who may review them [9]. The World Health Organisation advocates the use of shared electronic medical records and an intersectoral approach to reorient health systems to person-centered care [5]. In this study, the important role of the hospital pharmacist in reconciling medications was highlighted. While it is unknown how or why so many medication discrepancies occurred, better medication reconciliation in the community is an opportunity for reducing burden associated with medication error. Whether this can be easily performed by the GP through more frequent medication review, or whether there is a role for the community pharmacist or general practice nurse, may depend on the community and available resources.

This study was conducted at a single regional site; it is known that there are fewer GPs in rural towns per capita than in urban areas, and this area has an older population and a high rate of chronic health issues [10]. The rate of medication discrepancy was calculated from the number of referrals with at least one discrepancy present. As 6% of the sample did not contain any discrepancies, the overall rate of medication discrepancy is likely to be slightly lower. Nevertheless, that one-quarter of these were of moderate to extreme risk suggests there is considerable opportunity to improve patient health outcomes through better medication reconciliation in the community.

## 5. Conclusions

This paper presents clinically important data that can contribute to ongoing education, quality assurance and meaningful interaction between medical professionals. Improving medication reconciliation in the community may reduce the burden associated with preventable medication errors. Whether this is achieved by more frequent GP-led medication review or community-based pharmacist medication review may depend on the community and available resources.

## Figures and Tables

**Table 1:** Frequency and type of medication discrepancy.

Type of Discrepancy	Frequency	Percent
Omission	152	24.7
Inclusion	246	40.0
Exclusion	106	17.2
Dose error	54	8.7
Frequency error	33	5.4
Dosage and frequency omission	17	2.7
Medication type	6	1.0
Time	2	0.3
Total	616	100

**Table 2:** Consequences of discrepancies.

Severity of Consequence	Frequency	Percent
Insignificant	274	44.4
Minor	248	40.3
Moderate	86	14.0
Major	8	1.3
Catastrophic	0	0
Total	616	100

**Table 3:** Likelihood of occurrence.

Likelihood	Frequency	Percent
Almost certain	1	0.2
Likely	27	4.4
Possible	118	19.2
Unlikely	265	43.0
Rare	205	33.2
Total	616	100

**Table 4:** Risk rating.

Type of Discrepancy	Frequency	Percent
Extreme	8	2.1
High	83	13.5
Moderate	76	11.5
Low	449	72.9
Total	616	100
